# In quest of China sports lottery development path to common prosperity in 2035

**DOI:** 10.1371/journal.pone.0297629

**Published:** 2024-01-26

**Authors:** Yaping Yao, Bin Wan, Bo Long, Te Bu, Yang Zhang

**Affiliations:** 1 College of Physical Education, Hunan Normal University, Changsha, P.R. China; 2 Institute of Sports and Health Industry, HEHA CAT Fitness, Changsha, P.R. China; 3 Independent Person, Windermere, FL, United States of America; Cavendish University / Kyambogo University, UGANDA

## Abstract

**Objectives:**

The China sports lottery contributes to sports and welfare causes. This study aims to construct a macro forecasting model supporting its sustained growth aligned with Vision 2035.

**Methods:**

The modeling employed a distributional regression. Sales data of the China sports lottery from 2011 to 2022 were chosen as the response variable, alongside various macro- and event-level explanatory factors.

**Results:**

A gamma distribution best fit the data. In the stable model spanning 2011–2019, urbanization, population dynamics, and FIFA emerged as significant contributors (Chi–square p < 0.05) to the location shift parameter. These three factors retained their significance in the 2011–2022 shock model, where shock itself notably impacted sales (p < 0.001). Utilizing the shock model, we simulated the trajectory of the China sports lottery up to 2035. China’s demographics changes are poised to create structural headwinds starting in 2026, leading to an anticipated decline in sales driven by population shifts from 2032 onward. However, the FIFA effect is projected to continue fortifying this sector.

**Conclusions:**

Beyond offering original insights into the sales trajectory until 2035, specifically concerning new urbanization, negative population growth, and the FIFA effect, this macro forecasting framework can assist in addressing the policy priority of balancing growth with risk mitigation. We recommend policymakers connect market development with mass sports, potentially garnering a dual boost from the growing population of older consumers and the inherent benefits of a “FIFA (mass sports)” effect. A people-centered approach to the China sports lottery could significantly contribute to the long–range objectives of achieving common prosperity outlined in Vision 2035.

## 1. Introduction

The public lottery has roots in a the thousand-year-old gambling culture, morphing into a vital source of funding for social welfare [1]. Within the lottery domain, the modern sports lottery commands substantial consumer interest across diverse cultures and nations [2], pivotal in fueling public sports fundraising efforts. Consider the British Olympic team’s ascent post the Atlanta 1996 Summer Olympics, partially credited to the surge in the national lottery [3].

China’s sports lottery operates under the mantra of “from the people, for the people.” In 1994, the General Office of the State Council authorized five provinces (city) to initiate the China sports lottery, managed by the Ministry of Finance and the General Administration of Sport of China. Post-2001, these lottery funds extensively supported welfare causes, such as allocating 2.75 billion CNY from 2001 to 2008 for constructing and operating the Beijing 2008 Summer Olympics venues, which became sustainable Olympic legacies for Chinese society. The “Guiding Opinions of the General Office of the State Council on Accelerating the Development of the Sports Industry” in 2010 emphasized catering to diverse sports consumption while enhancing livelihoods [4]. This top-level design steered the comprehensive sports industry development. Subsequent initiatives like the “13th Five-Year Plan for Sports Lottery Development” underscored expanding issuance scope and elevating development quality [5]. The 2019 “Outlines for Building a Sports Power” marked the sports industry’s position within China’s long-range economic model, urging the expansion of sports lottery funds for national fitness events [6]. These policies facilitated the rapid growth of the China sports lottery. Sales between 2012 and 2022 totaled 2,198.6 billion CNY, generating 553 billion CNY for sports lottery public welfare funds [7], robustly financing Chinese sports and social initiatives. These achievements highlight the pivotal role of the China sports lottery in supporting socialist causes. Yet, its future must align with public aspirations for an improved standard of living, extending beyond economic metrics.

The Organisation for Economic Cooperation and Development’s 2018 publication, “Beyond GDP” [8], challenges the sole reliance on GDP to gauge national development’s overall “health.” Stiglitz et al. argue that beyond a certain GDP per capita, individuals’ well-being does not linearly progress [9], extending this concept to societies and countries collectively. To address this, they propose non-monetary well-being indicators for a more holistic assessment of social progress to guide policies. Similarly, we posit that China’s sports prowess should not singularly define its status as a dominant power. Instead, the sports sector’s primary contribution lies in advancing public health, the cornerstone of the Chinese path to modernization [10]. Shifting ideologies to prioritize alternative national developmental indicators, like population well-being, raises a crucial question for the future of the China sports lottery: Will its sales authentically reflect ethos of “from the people, for the people”?

For policymakers, we believe the answer to this question rests on two fundamental aspects. On one hand, the China sports lottery serves as a significant source for augmenting welfare activities. The funds allocated toward public sporting facilities, disability causes, and reducing the infrastructural disparities between urban and rural areas undeniably contribute to national development. Therefore, its sales will remain a crucial indicator of its sustainable growth. On the other hand, it is undeniable that sports betting can be addictive and have detrimental effects on an individual’s mental health [11]. From this perspective, unregulated market expansion, characterized by excessive involvement of key sociodemographic groups such as the youth, may lead to increased sports lottery sales, but arguably poses detrimental consequences for Chinese society in the long run. As far as our knowledge extends, neither researchers studying the China sports lottery nor the relevant regulatory bodies have publicly acknowledged this concern, which contradicts China’s common prosperity policy.

The concept of common prosperity has been integral since the establishment of New China [12], but it is under Xi Jinping’s leadership that significant efforts were made to eliminate income inequities resulting from the early developmental model, particularly in terms of urban–rural, regional, and social disparities. The 14th Five-Year Plan outlines that “By 2035, China will have basically achieved socialist modernization” and one of the long-range objectives is “Our people will lead even better lives, with more substantive advances in people’s well-rounded development and common prosperity” [13]. Accordingly, common prosperity, aiming to reduce developmental disparities and promote well-rounded human development, is the essential goal of China’s approach to modernization. Therefore, any efforts to promote development must align with Vision 2035. A healthy growth of the China sports lottery has the potential to support modernization efforts, but a reckless expansion of this industry that encourages widespread gambling conflicts with the people-centered development philosophy. Hence, it is imperative to analyze the historical growth pattern of the China sports lottery to formulate a successful developmental path toward common prosperity.

In the latest 14th Five-Year Plan for Sports Development, the government explicitly guides that, “The development of sports lotteries should simultaneously improve in terms of efficiency and quality, adhere to the positioning of national public welfare lotteries, strengthen the management of sports lotteries, enhance the level of legalized lotteries, and improve the long-term mechanism for the prevention and control of risks in sports lotteries” [14]. Consequently, policymakers acknowledge both the significance of ongoing growth of the China sports lottery and the necessity to mitigate potential adverse consequences in this pursuit. Therefore, there is a strong need for research that addresses both these issues comprehensively. However, none of the existing research conducted so far has adequately addressed both concerns, especially within the overarching context of Vision 2035. To support the all-round and high-quality development of the Chinese path to sports modernization and Vision 2035 [10], this study endeavors, for the first time, to model and forecast the sales of China sports lottery through 2035. The proposed strategies will align closely with the people-centered philosophy and aim to expand market development for both improved social welfare and the promotion of a healthy sports culture.

## 2. Literature review

To predict the sales of the China sports lottery, it is crucial to identify potential influential factors. Here, a macro perspective gleaned from both domestic and international markets can offer valuable insights. The sports lottery largely aligns with mass socioeconomic tendencies for entertainment, mindfulness, and personal gain. Consequently, population concentration—nowadays, urbanization—often precedes market growth. Walker and Jackson, using state-level data from 1985 to 2000, observed that major urban areas in the United States yielded more legal gambling revenues and tax collections than less populous regions [15]. This clustering effect in urban settings partly owes itself to the total locations and density of lottery participation [16], as lottery sales inherently hinge on these factors. China’s urban population has continually risen since the era of reform and opening-up, with the urbanization rate reaching 65.22% in 2022 [17]. The influx of rural populations into cities does not solely comprise workers but also new consumers, vital for the sports industry’s high-quality development. Spatial analysis has revealed significant clustering in sales of the China sports lottery sales [18]. Fang and Chen, using panel data from 31 provinces and municipalities spanning 2007 to 2016, established a causal link between the urbanization rate and sports lottery consumption [19]. Similarly, China’s inter-provincial panel data bolster the correlation between the density of sports lottery betting machines and sales [20]. Therefore, rapid urbanization has remained a key driver of the population-density-lottery sales nexus and is expected to continue until urbanization reaches saturation.

In the Chinese context, long-term urbanization correlates with increased economic development, higher GDP per capita, and a more educated population [21], with the latter two being dynamic albeit less straightforward factors in lottery sales. Various countries [22], including China [23], present compelling evidence that low-income groups tend to participate more in speculative lottery activities, while high-income groups often seek returns from alternate sources like long-term equity investments and career advancement. As personal economic resilience strengthens, the correlation between GDP per capita and sports lottery sales tends to diminish. In China, this threshold has been identified at approximately 32,538 CNY, beyond which sales of the China sports lottery decline [24]. Despite the existence of economic status-based speculative behaviors, the elasticity of GDP per capita, as an outcome of economic growth, serves as a lagging but long term positive factor for sports lottery sales [25].

In general, less-educated groups display a higher inclination to test their luck for uncertain financial gains [26]. This socioeconomically-driven psychology in human behavior, albeit regrettable in our view, has been consistently observed in both highly developed [27], frontier [28] economies, and China [20]. Over time, particularly owing to the consistent improvement in income and education, the perception of public welfare among higher-income Chinese individuals continues to rise, leading them to participate in sports lotteries for social causes and values [29].

A recent industrial survey spanning 2018 to 2020 indicates a notable trend among users of the China sports lottery mobile app, increasingly favoring the under-34 age bracket. Remarkably, 96.8% of lottery consumers, encompassing the welfare lottery as well, fall under the age of 44 [30]. This demographic shift holds significant implications for the future. First, this younger age cohort typically undergoes compulsory high school education, with a substantial proportion of adults born after 1995 holding college degrees. Given the government’s focus on comprehensive education outlined in Vision 2035, which aims to provide lifelong learning opportunities for all in modern Chinese society, the future consumers of the China sports lottery will likely comprise a more educated populace with greater economic parity, potentially diminishing the relevance of this sociodemographic factor. Second, a robust economy relies on its population foundation, and China has benefited from a demographic dividend for over half a century. As the population ages, Chinese society anticipates considerable challenges in the coming year [31]. The established correlation [32] between total population and lottery sales emphasizes the significant role demographic dynamics play in economic activities, including sports lottery sales [33].

In econometrics, a comprehensive evaluation of economic growth often factors in inflation or M2 supply, which, to our knowledge, has not been addressed in this field of research. The impact of inflation is intricate. In stable macro environments, evidence from the housing sector suggests that market expansion aligns with monetary growth [34]. Conversely, recent findings from the United States indicate that persistent inflation negatively impacted sports betting [35], hinting at a potential inverse-U-shaped relationship between inflation and market growth. Given the absence of existing theories, particularly concerning the sales of the China sports lottery, exploring this factor could prove insightful.

While factors like the number of million-CNY prizes [20], the marketing costs [36] exhibit some influence on lottery sales, their macro relevance appears relatively lesser. Nonetheless, these findings contribute to a solid theoretical foundation. Building upon these critical discoveries, this study endeavors to forecast China sports lottery sales until 2035, aiming to serve as a policy reference and preparation for an evolving socioeconomic landscape in transition.

## 3. Materials and methods

### 3.1. Variables

The data supporting this study’s conclusions are accessible on figshare (DOI: https://doi.org/10.6084/m9.figshare.23826753.v1). The study focuses on the sales of the China sports lottery from 2011 to 2022. The inception of top-level design in the sports industry began directing policies in 2010, coinciding with numerous national policy enactments [37]. This era encompasses the 12th and 13th Five-Year Plans, marking not only a rapid developmental phase in the Chinese path to sports modernization [10] but also a defining period for the Chinese economy since the reform and opening-up.

**Fig 1:**
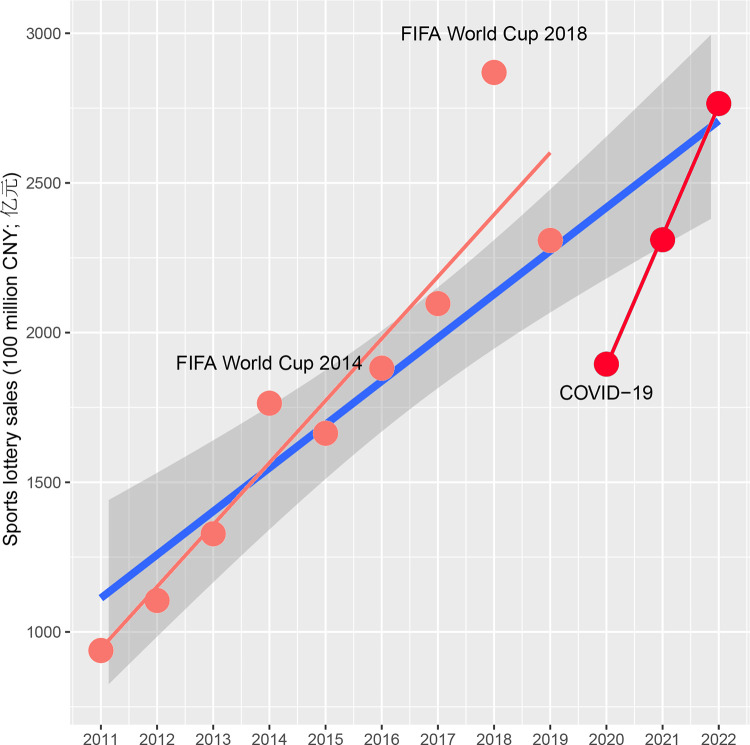
Sales of China sports lottery between 2011 and 2022. The pink line indicates the trend between 2011 and 2019, the red line indicates the trend between 2020 and 2022, and the blue line indicates the overall trend within the data set. The confidence level (shaded region) for the overall data is set at 90%.

Fig 1 illustrates the sales of the China sports lottery from 2011 to 2022, revealing a consistent growth trend amid years marked by heteroscedasticity. The data structure suggests that the China sports lottery is influenced not only by endogenous mechanisms but also by exogenous events, reflected in the tail’s scale. This behavior exhibits a dual nature. First, the FIFA World Cup had an asymmetrically positive impact on sales, displaying a potentially increasing effect in subsequent years. Consequently, the FIFA World Cup was considered a factor in modeling, with the response variable coded based on the presence or absence of the tournament in a given year. Second, the onset of the global pandemic in 2020 significantly impacted the data. Given the intermittent halting of global socioeconomic activities, the data for 2020 was designated as a shock, representing this global-scale event. The ripple effects of COVID-19 continued to influence the Chinese economy until 2022, with a particularly regional impact noted in that year. As such, the 2020 data was coded as a global shock, the 2022 data as a domestic shock, and the remaining data as having no shock. Consequently, two models were employed to analyze the negative tail event further.

Additionally, this study examined four quantitative macro variables: urbanization rate (in percentage), population aged 15–64 years (in 10,000 people), GDP per capita (in CNY), and inflation rate (in percentage). Inflation measurement choice was influenced by Blagrave and colleagues’ suggestion that the GDP deflator for emerging markets provides a more accurate indicator of overall economic changes than the CPI [38]. This study approximated the GDP deflator by subtracting the real GDP growth rate from the nominal GDP growth rate in corresponding years due to China’s lack of published real GDP data based on previous year’s constant prices. In total, this study models the sales of the China sports lottery using two event-type variables (i.e., FIFA and two magnitudes of shock) and four macro-level variables. Historical data were collected from the China Statistical Yearbook, the China Lottery Yearbook, and the official press release from the National Bureau of Statistics of China.

### 3.2. Modeling

This study employed a generalized additive model for location, scale, and shape framework [39] to handle the data structure, which includes both quantitative and categorical predictors. This distributional regression approach is renowned for its flexibility in exploring data without pre-existing theoretical assumptions. Notably, it has been utilized by prominent institutions such as the IMF for bank stress tests and the WHO for current child growth standards. Interested readers are directed to Stasinopoulos and Rigby’s original work for a comprehensive understanding of the theory [40].

Statistical analyses were performed using the gamlss package (version 5.4–12) in RStudio (version 2023.03.0 Build 386). The Akaike information criterion (AIC) [41] was selected as the benchmark for evaluating goodness-of-fit. The rationale behind this choice is that while the Schwarz Bayesian Criterion could marginally alter the penalty κ from 2 to 2.48, it does not significantly affect the current distribution fitting. Additionally, considering future model refitting, for instance, in 2030, the κ based on the Schwarz Bayesian Criterion would likely be 2.996, indicating minimal deviation from the AIC. Hence, for simplicity and consistency, the AIC was adopted for model selection.

As multiple terms (i.e., explanatory variables) were expected in the conditional model, potentially displaying a multiplicative effect, we anchored the log link function for the location parameter, while various link functions (e.g., log, inverse, identity) were assessed for the scale parameter. Over 40 explicit and generated distributions (e.g., log-transformed, left-truncated) were evaluated to fit the response variable, and the distribution suitably linked with the minimum AIC value was selected as the marginal distribution. For term selection, we initially fitted a null model encompassing only the intercept. Subsequently, we employed a stepwise strategy (refer to Ramires and colleagues’ work for comprehensive details) [42]. Determination of the model term’s significance was carried out using the generalized likelihood test, generally preferable over the Wald test statistic. The data structure indicated that linear terms sufficed for modeling, obviating the need for hyper-parameter selection in this study. Finally, verification of the model residual was conducted using the worm plot [43], a detrended QQ plot.

We executed the modeling procedures twice—once for the 2011–2019 period and once for the entire dataset. Given the magnitude of the 2020 shock and its enduring impact, our interest lay in assessing whether the long-term endogenous mechanisms underwent any change. Thus, we provided two models: a stable model representing the 2011–2019 range and a shock model spanning 2011–2022, aiming to aid future decision-making and inference.

## 4. Results

### 4.1. Stable model

Table 1 summarizes the selected model terms, providing the generalized likelihood ratio test statistic and its associated Chi-square p-values. Based on the AIC, the inverse gamma model (AIC = 91.848) would seemingly be the optimal choice. However, the gamma model merely differs by 0.672 units in AIC (model 1 in Table 1). In practical terms, the location parameter of a gamma distribution signifies the mean, whereas the scaling parameter denotes the coefficient of variation. The gamma distribution, with greater interpretability advantages over the more complex inverse gamma, was chosen in the conditional model, adhering to the principle of parsimony.

**Table 1:** Sales of China sports lottery modeled with GAMLSS distributions.

Term	Model 1		Model 2		Model 3	
	χ^2^	p	χ^2^	p	χ^2^	p
***μ***						
**urbanization**	30.886	2.737e-08	54.263	1.754e-13	60.054	9.231e-15
**population**	22.825	1.774e-06	32.484	1.202e-08	29.999	4.323e-08
**FIFA**	33.523	7.043e-09	32.588	1.139e-08	34.907	3.459e-09
**shock 1**	-	-	29.726	4.976e-08	-	-
**shock 2**	-	-	-	-	15.000	0.000553
***θ***						
**GDP per capital**	7.2746	0.006994	-	-	-	-
**shock 1**	-	-	25.216	5.124e-07	-	-
**AIC**	92.519		109.837		133.464	

**Fig 2:**
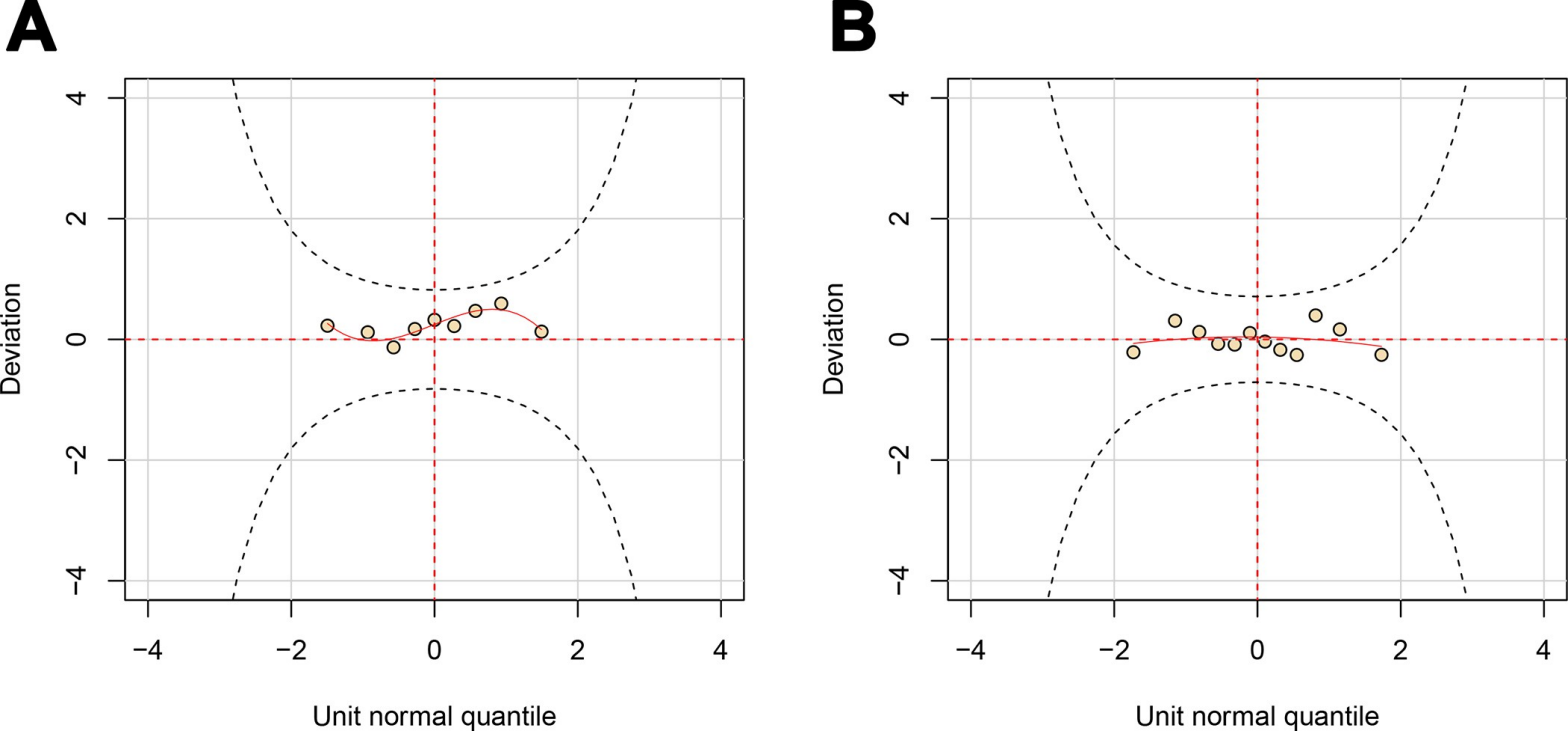
Worm plot of the model residual: (A) stable model; (B) shock model.

The worm plot (Fig 2A) of the conditional model reveals two patterns. First, all points fall between the two elliptic curves, indicating model support for the response variable. Second, the points’ positioning above the horizontal line suggests an overestimation of the mean in the residual. From a modeling perspective, this indicates an underestimation of the location, warranting adjustment (e.g., modifying link function, altering distribution).

However, as this model is solely employed for qualitative interpretation, we adopt it as the stable model, outlined in Eq 1:

$$\begin{array}{c} Y_{stable}=GA(\hat{\mu },\;\hat{\sigma })\\ \hat{\mu }=\exp[‐14.05\;(SE,\;0.9634)+0.08798\;(SE,\;0.002099)\times urbanization+0.1815\;(SE,\;\\ 0.007229)\times FIFA\;World\;Cup\;(if\;yes)+0.0001627\;(SE,\;0.00001053)\times population]\\ \hat{\sigma }=\exp[‐11.88\;(SE,\;1.141)+0.0001452\;(SE,\;0.00002158)\times GDP\;per\;capita] \end{array}$$ (1)

where SE is the standard error.

In the stable model, the urbanization rate, population aged 15–64 years, and the FIFA World Cup emerged as significant predictors of the location shift parameter (model 1 in Table 1). These variables positively contributed to China sports lottery sales between 2011 and 2019. Only GDP per capita exhibited a significant effect on the scaling parameter (model 1 in Table 1), indicating that an increase in GDP per capita was associated with increased variation.

### 4.2. Shock model

The gamma distribution was selected to model the data spanning from 2011 to 2022. Within the single shock model (model 2 in Table 1), only the year 2020 is encoded, whereas in the dual shock model (model 3 in Table 1), both global-level and domestic-level shocks are accounted for. Initially, the Chi-square p-values indicate statistical significance for both shock codings. However, after employing a stepwise procedure, only the global-level shock remained significant in model 3. Consequently, both shock models indicate that the global pandemic in 2020 significantly impacted sales. Model 2 demonstrates superior goodness-of-fit compared to model 3, as evident from the AIC (Table 1). Further examination of residual behavior (Fig 1B) confirms that model 2 meets the modeling criteria. The resulting shock model, based on the gamma distribution, is presented in Eq 2:

$$\begin{array}{c} Y_{shock}=GA(\hat{\mu },\;\hat{\sigma })\\ \hat{\mu }=\exp[‐6.188\;(SE,\;0.6864)+0.09127\;(SE,\;0.002295)\times urbanization+0.00008288\\ (SE,\;0.000005821)\times \mathrm{population}+0.2149\;(\mathrm{SE},\;0.01534)\times FIFA\;World\;Cup\;(if\;yes)‐\\ 0.1254\;(\mathrm{SE},0.01477)\times \mathrm{shock}\;(\mathrm{if}\;\mathrm{yes})]\\ \hat{\sigma }=\exp[‐3.8354\;(SE,\;0.2132)‐12.5105\;(SE,\;0.7373)\times shock\;(if\;yes)] \end{array}$$ (2)

where SE is the standard error.

This shock model highlights the significant contributions of the urbanization rate, the population aged 15–64 years, and the FIFA World Cup to the sales of the China sports lottery between 2011 and 2022. As anticipated, the global event in 2020 also exhibited a substantial impact on sales. According to the model, the variation was adjusted by the N = 1 event.

### 4.3. Forecasting

Utilizing this equation, it becomes feasible to simulate the sales of the China sports lottery. The shock model requires the input of two quantitative variables and two categorical variables for the location shift parameter. Concerning the urbanization rate, predictive values sourced from the Chinese Academy of Social Sciences [44] were utilized. The research titled “Forecast of urbanization level under the goal of integrated urban–rural development” presented two scenarios projecting urbanization until 2035. The first scenario predicts a reduction in the urban–rural income gap to 1.8, resulting in a projected urbanization rate of 75.4% by 2035. In the second scenario, with an urban–rural income gap projected at 2.0, the anticipated urbanization rate is 74.7%. A balanced interpretation was achieved by averaging these scenarios. Regarding the estimated population aged 15–64 years, projections from the YuWa Population Research [45] were consulted. For obvious reasons, predicting and hedging a global event in 2020 was not realistic, and thus this factor was excluded from our forecasts (set to zero).

**Fig 3:**
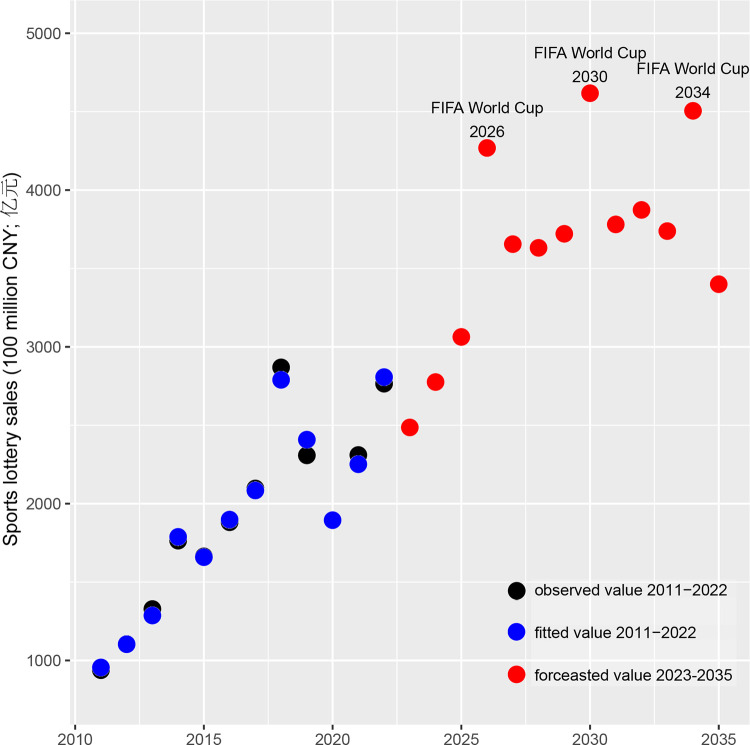
Forecasted sales of China sports lottery through 2035.

Considering this context, Fig 3 illustrates the simulated developmental trajectory of the China sports lottery. The projection suggests that China’s population aged 15–64 years will peak in 2026 and subsequently decline by 69.09 million by 2035. Without factoring in the impact of the FIFA World Cup, this demographic transition predicts a plateau in sales from 2027 onward, followed by a decline starting in 2032. Notably, the projected urbanization rate [44] in 2021 was 0.6% lower than the baseline, resulting in an underestimation of sales in our forecast.

## 5. Discussion

Employing a distributional regression approach, this study models the historical factors influencing the sales of the China sports lottery. The resulting models not only offer analytical power to elucidate the inherent mechanisms but also demonstrate a reasonable capacity for forecasting prospective developmental trajectories. Past observations revealed a confluence of factors: urban expansion, a robust population base, and an overall favorable macro trend characterized by globalization and low unemployment. These elements collectively bolstered the solid development of the Chinese economy, including the China sports lottery. While the urbanization trend is anticipated to persist, potential disruptions from global macro shifts (e.g., unionization) and concerning internal demographic structures may impede future economic growth. Bearing these experiences and future visions in mind, each significant factor identified in our models stands as an important guide for the China sports lottery’s development toward sustainable, long-range objectives.

A remarkable aspect is the consistency and resilience of market development. Despite the 2020 crisis, the overall trajectory swiftly reverted to its long-term trends, a phenomenon observed across various sectors of the Chinese economy. This rebound is attributable to the spatial agglomeration strategy in Chinese urban development [46]. China’s new-type urbanization serves as a composite indicator, reflecting not just urban population density but also the socioeconomic dimensions of modern human activities. As urbanization progresses, the agglomeration and scale effects of cities expand, optimizing the consumption environment, concepts, and trends, thereby exponentially driving market demand. This trend is evident in both stable and shock models, showcasing the high impact of the urbanization rate as a predictor of sales, not only in the China sports lottery but also across other sectors of China’s modern economic system [19, 47].

A spatial analysis of regional sports lottery data underscores the persistence of market development heterogeneity [48], indicating untapped potential for expanding sales in the western and northern regions. The western region, characterized by large populations and a low urbanization rate, necessitates a policy focus on modernization. This modernization drive in inland regions aligns with China’s second centennial goal, emphasizing the importance of achieving common prosperity. Correspondingly, the populous southwestern region, with its robust population-economic activity nexus and relatively low-education levels, may significantly drive regional lottery development and the China sports lottery as a whole. Regarding urbanization rates, the modernization of inland regions and the March Westward strategy [49] might initially underestimate and later overestimate sales in our model. This shift occurs as large-scale infrastructure constructions near completion, causing a decline in the relative marginal benefit of urbanization investments. It is essential to bear in mind the statistical adage that “all models are wrong but some are useful [50].” While many macro environments continually evolve, the modeling principles outlined in this work remain relevant. Like most econometric models for policy reference, forecasting models linking “y” to “x” should be regularly updated with incoming data to enhance precision.

While engaged in this study and formulating policy recommendations, we uphold a people-centered approach: fostering the healthy, synchronized development of the market and less privileged social classes, and pursuing long-term goals for a stronger sports nation and common prosperity across social strata. While it is undeniable that sports lottery public welfare funds contribute to modernizing Chinese society, we challenge assertions such as the view of lottery funds as a third distribution function, in addition to market distribution and tax redistribution—a pivotal governance tool [20]. Sociological research definitively illustrates that low-income, low-education groups tend to engage in speculative behavior, hoping to improve their socioeconomic status [51]. When economists propose the lottery as an additional income transfer channel [52], and policymakers entertain such theories without caution, they may underestimate the impact of betting on the psychological pursuit of genuine Chinese values among the less privileged. Consequently, this could widen the inequality gap. Market opportunists might exploit their eagerness for change and lack of comprehension regarding harmful addictive behavior [53]. Recent reports even highlight illegal lottery activities infiltrating Chinese elementary school campuses.

Considering these points, two factors stand out in our models for fostering healthy, synchronized development in the market and society, aligning with Healthy China 2030 and Vision 2035. Our models highlight the asymmetric impact of the FIFA World Cup on lottery sales, underscoring soccer’s popularity and FIFA’s economic influence. Notably, data from non-FIFA World Cup years show that sales from competitive sports lotteries (e.g., bets on Chinese Super League games) constitute a significant portion of the overall China sports lottery, indicating the rising popularity of soccer betting in China. Practically speaking, the lottery market operates separately from the real economy despite accumulating substantial amounts, with annual sales from competitive sports lotteries often exceeding 100 billion CNY. Essentially, it fuels entertainment and speculative economic activities but does not significantly contribute to building a robust sports nation or a healthy populace, as envisioned in governmental policies. We believe this demands two focal points for policymakers’ market development strategies by 2035. First, there exists a delicate balance between entertainment and addiction. International experiences suggest that unsupervised soccer betting among youth can lead to a social crisis [54]. Consequently, policymakers should reassess current participation rates in competitive sports lotteries and consider implementing restrictive measures—like setting total bet amounts and frequencies for different age groups—to strike a balance between entertainment and the risk of addiction.

Second, a refined competitive sports lottery could establish a distinct connection between widespread sports participation, a healthy populace, a robust sports nation, and the tangible economy. Psychological insights into gambling indicate diverse motivations beyond mere betting, including social interaction [55]. A defining feature of the Chinese path to sports modernization is its extensive promotion of sports at the grassroots level [10]. Through broad sports engagement, individuals from varied socioeconomic backgrounds could attain mindfulness and nurture social cohesion [56]. Drawing from the “FIFA effect,” it can be inferred that consumers seek a participatory experience. Should such an experience be integrated into mass sports where consumers actively participate, it would be intriguing to explore if our design could encourage a sustained, healthy lifestyle change involving regular physical activity. Consumers would increasingly recognize the welfare aspect of the China sports lottery, actively engage in sports activities across all levels, and experience the positive flow of contributing to a more egalitarian society, mindfulness, and physical well-being. Leveraging the halo effect [57] by allocating a larger share (e.g., 50% of lottery sales in a specific mass sport) and ensuring transparency in the utilization of sports lottery public welfare funds [58] could empower this innovative model of the China sports lottery. This approach has the potential to establish a novel consumption pattern linked to the actual economy and align with the long-term goals outlined in Healthy China 2030 and Vision 2035.

It is easy to conflate an aging society with diminished economic dynamism. As individuals age, their consumer behavior tends to become more rational. Yet, beyond medical needs, older adults still aspire for aspects like healthy aging and social interaction. To sustain economic engagement within this demographic, innovative consumption strategies are imperative. As part of the emerging demand-side consumption trend, policymakers could orchestrate mass sports activities for older adults and promote low-cost, entertainment-oriented sports lotteries to facilitate socialization. Through sports modernization, an aging yet healthy society can continue contributing to the vigor of China’s future economy. This strengthens the belief that future consumers of the China sports lottery will encompass a robust sports populace across all age groups, supporting a resilient sports nation and fostering a healthier China.

## 6. Conclusions

In contrast to other sectors within the sports industry, the evolution of China’s sports lottery holds the potential for both favorable economic growth and various degrees of social progress—either positive or negative. Effectively regulated market development will play a pivotal role in fostering a system aimed at benefiting and serving the public. Conversely, unregulated expansion may lead to excessive involvement of young individuals in sports gambling, potentially fostering addiction and detrimentally impacting the nation’s overall competitiveness in development. China currently stands at a critical juncture on its path toward achieving its second centennial goal, where the attainment of common prosperity by 2035 serves as a key metric to assess socioeconomic progress

Given these considerations, this study initiates by constructing a macro forecasting model for the China sports lottery up to 2035. Employing a GAMLSS modeling approach, our forecast identifies three interconnected dynamics in the sales trajectory leading up to 2035. First, our model suggests that sustained urbanization will continue to underpin market expansion. Projections indicate a maintenance of current growth rates until 2027, followed by a gradual slowdown. Thus, high-quality urbanization, as defined in the latest government strategy, will remain a fundamental driver for the China sports lottery and the broader sports industry over the next nine years [59]. During this period, policymakers in the China sports lottery sphere can serve both as beneficiaries and contributors. Second, another significant driver of past market growth—the population aged 15–64 years—will begin to diminish as early as 2026, resulting in a gradual decline in the consumer base. Projections foresee 2032 as a turning point, where sales are expected to steadily decrease without the addition of new consumers. This demographic shift necessitates proactive strategies from policymakers, such as increasing the participation rate of older adults, to counteract this demographic headwind. Third, our analysis unmistakably highlights the potential impact of events like the FIFA World Cup as momentary catalysts for market growth. Strategically promoting sports lottery alongside major events like the FIFA World Cup and competitive sports leagues (e.g., the Chinese Super League) could bolster market growth in terms of economic returns. However, the development of the China sports lottery could be more beneficial for Chinese society as a whole. Our recommendation is for policymakers to integrate sports lottery initiatives with mass sports at all societal levels in China. This approach not only mimics the effects of a year-long FIFA World Cup but also encourages lottery consumers to become active participants in sports, thereby benefiting from regular physical activity. Ultimately, the nation stands to gain long-term advantages from a healthier population across all age groups, fostering a robust economy and promoting common prosperity across social classes in China. In summary, these findings offer valuable policy insights for nurturing sustainable growth within the China sports lottery amid a declining population trend. The methodology presented in this work remains adaptable to accommodate new macro developments and can be utilized to refine policy directions.

According to these findings, the development of two pivotal theoretical frameworks is essential for fine-tuning future policy decisions on a micro-level. First, population aging presents itself as a symptomatic facet of urban contraction. Since 1949, modern Chinese society has thrived on an unparalleled level of productivity. However, there exists no historical precedent for the swift aging of society and its consequential impact on consumer behavior, both within China and globally. While extensive research has delved into the general effects of population aging on consumption [60], limited focus has been directed specifically toward its influence on sales within the China sports lottery [33], let alone the formulation of viable strategies to address this issue. Our notion of integrating the China sports lottery with mass sports remains theoretical, albeit rational. Given the challenges involved, it warrants an in-depth exploration of the following topics: (1) While grassroots sports are prevalent across all levels and age groups within Chinese society, there is a noticeable scarcity of organized mass sports tailored for older adults. Consequently, establishing a fiscal support mechanism for senior sports organizations poses an initial obstacle in our strategy. (2) As previously mentioned, various tangible consumption attributes (such as the socializing aspect) and intangible factors (like price sensitivity) significantly influence the support of older adults for the China sports lottery. Thus, it is imperative for studies in cultural consumerism [61] to investigate these attributes and devise customized China sports lottery options for this demographic. Second, despite an abundance of international research on strategies to curb unregulated sports gambling among young individuals [62], these approaches might offer limited insights within the Chinese context. China has a longstanding gambling culture, and individuals with insufficient financial literacy are particularly susceptible to engaging in speculative activities. The retail frenzy observed during the 2015 Chinese stock market boom exemplifies this cultural inclination. Therefore, consumer research should promptly redirect its focus toward formulating policy strategies to regulate the involvement of Chinese youth in competitive sports lotteries [63].

Finally, this study did not incorporate the national unemployment rate into its model, as it remained stable within an acceptable range (between 4 and 5 percent) up to 2020. However, panel data at the provincial level indicate that the regional unemployment rate had both a direct positive effect [64] and a positive spillover effect [18] on lottery sales. The phenomenon of rising unemployment leading to riskier financial choices, such as gambling, has also been observed in other countries [65]. In the 2020s, macroeconomic volatility has become more unpredictable, with the youth unemployment rate in China reaching 20% in the first half of 2023. If employment opportunities do not improve, coupled with geopolitical tensions and the psychological impact from generative AI, the unemployment rate might paradoxically trigger surges in China sports lottery sales. We caution that this trend could be detrimental to sustainable market growth in the long term, impacting the national economy and overall competitiveness.
